# Metabolic profiling and enzyme inhibitory activity of the essential oil of *citrus aurantium* fruit peel

**DOI:** 10.1186/s12906-024-04505-2

**Published:** 2024-07-10

**Authors:** Naglaa S. Ashmawy, Nilofar Nilofar, Gokhan Zengin, Omayma A. Eldahshan

**Affiliations:** 1 Pharmacognosy Department, Faculty of Pharmacy, Ain Shams University, Cairo, 11566 Egypt; 2 Department of Biology, Science Faculty, Selcuk University Campus, Konya, Turkey; 3 Department of Pharmacy, Botanic Garden “Giardino dei Semplici”, Università degli Studi “Gabriele d’Annunzio”, via dei Vestini 31, Chieti, 66100 Italy; 4 Center of Drug Discovery Research and Development, Ain Shams University, Cairo, 11566 Egypt; 5 Department of Pharmaceutical Sciences, College of Pharmacy, Gulf Medical University, P.O. Box 4184, Ajman, United Arab Emirates

**Keywords:** *Citrus aurantium*, Essential oil, GC-MS, Antioxidants, Enzyme inhibition

## Abstract

**Background:**

Bitter orange (*Citrus aurantium*) is a fruiting shrub native to tropical and subtropical countries around the world and cultivated in many regions due to its nutraceutical value. The current study investigated the metabolic profiling and enzyme inhibitory activities of volatile constituents derived from the *C. aurantium* peel cultivated in Egypt by three different extraction methods.

**Methods:**

The volatile chemical constituents of the peel of *C. aurantium* were isolated using three methods; steam distillation (SD), hydrodistillation (HD), and microwave-assisted hydrodistillation (MAHD), and then were investigated by GC-MS. The antioxidant potential was evaluated by different assays such as DPPH, ABTS, FRAP, CUPRAC, and phosphomolybdenum and metal chelating potential. Moreover, the effect of enzyme inhibition of the three essential oils was tested using BChE, AChE, tyrosinase, glucosidase, as well as amylase assays.

**Results:**

A total of six compounds were detected by GC/MS analysis. The major constituent obtained by all three extraction methods was limonene (98.86% by SD, 98.68% by HD, and 99.23% by MAHD). Differences in the composition of the compounds of the three oils were observed. The hydrodistillation technique has yielded the highest number of compounds, notably two oxygenated monoterpenes: linalool (0.12%) and α-terpineol acetate (0.1%).

**Conclusion:**

In our study differences in the extraction methods of *C. aurantium* peel oils resulted in differences in the oils’ chemical composition. *Citrus* essential oils and their components showed potential antioxidant, anticholinesterase, antimelanogenesis, and antidiabetic activities. The presence of linalool and α-terpineol acetate may explain the superior activity observed for the oil isolated by HD in both radical scavenging and AChE inhibition assays, as well as in the enzyme inhibition assays.

## Introduction

The *Citrus* genus (Rutaceae) comprises approximately 140 genera and 1300 species [1]. This diverse group of tropical fruits includes well-known species like orange (*C. sinensis*), mandarin (*C. reticulata*), limes (*C. aurantium*), lemon (*C. limon*), and grapefruit (*C. paradisi*) [2]. *Citrus* fruits are not only delicious but also include nutritional value, containing significant levels of bioactive compounds, including citric acid [3] flavonoids [3, 4], limonoids [5], essential oils (EOs), vitamins, especially vitamin C and carotenoids [6] and alkaloids [7] all of which are responsible for various health benefits. Numerous studies have demonstrated the diverse health benefits and biological activities of *Citrus* and its constituents [8]. Additionally, different parts of the *Citrus* plant, including the leaves, flowers, fruits, and peels, are rich sources of essential oils. *Citrus* EOs have been reported for their antioxidant, anticancer [9], antibacterial [10], antidiabetic [11], insecticidal [12], and antifungal [13]. These properties have significant applications in various industries, including cosmetics, agriculture, food production as well as pharmaceutical formulations [14, 15]. *C.aurantium* species known as bitter orange is a fruiting shrub native to tropical and subtropical countries around the world and cultivated in many regions due to its nutraceutical value as well as economic importance [16]. Several studies reported the biologically active essential oils extracted from peels, leaves (petitgrain), and flowers (neroli) of *C*. *aurantium* [17–20]. Peel volatile oil particularly has been notified in literaturefor its significant economic value and market demand, being used in flavor, fragrance, medicine, and other fields [21]. In our previous study [23], we investigated the chemical profile of *C. aurantium* leaf oil that was extracted by 3 different methods as well as its biological *in-vitro* activities. However, despite the existing knowledge on the biological activities of Citrus EOs, there remains a need to further explore and compare the antioxidant and enzyme inhibition activities of peel EOs extracted through different methods, which is the primary focus of this study.

## Experimental

### Plant collection

The fruits of *C. aurantium* were obtained in March 2023 from Shibin Al Kawm City in a private garden, Al Minufiyah, Egypt. Authentication of the plant was done by Dr. Usama K. Abdel Hameed, Department of Botany, Faculty of Science, Ain Shams University, Cairo, Egypt. A voucher specimen under the code PHG-P-CA-461 was preserved in the Herbarium of the Department of Pharmacognosy, Faculty of Pharmacy, Ain Shams University, Cairo, Egypt.

### Essential oil preparation

#### Hydrodistillation

Essential oil was extracted from the dried peels of *C. aurantium* by hydrodistillation by Clevenger-type glass apparatus for four hours with a solid-liquid ratio of 250 g/800 mL. The extracted oil (1.20 mL) was preserved in sealed dark vials at 4 °C. A triplicate of the experiment was conducted.

#### Steam distillation

*C. aurantium* (dried peels, 250 g) was subjected to steam distillation for 3 h using the apparatus of steam distillation. The oil (1.00 mL) was dried using Na_2_SO_4_ (anhydrous) and stored in a sealed dark vial at 4 °C. A triplicate of the experiment was conducted.

#### Microwave-assisted hydro-distillation

The dried peels of *C. aurantium* (250 g) were homogenized and soaked in distilled water (500 mL) and placed for 35 min. in the microwave at the radiation of 80% to yield 0.7 mL of the extracted essential oil.

### GC/MS analysis of essential oils

The essential oil compositions obtained from the three isolation procedures were examined using GC coupled with MS. The Shimadzu GC/MS-QP 2010 (Koyoto, Japan) was connected to a mass spectrometer (SSQ 7000 quadrupole: Thermo-Finnigan, Bremen, Germany). A capillary column (model number Rtx-5MS, length 30 m, internal diameter 0.25 mm, film thickness 0.25 μm; Restek, USA) was utilized. After two minutes of gentle temperature adjustment to 45 ℃, the temperature was increased to 300 ℃ (rate of 5℃/minute for 5 min). A temperature of 250 ºC was maintained for the injector and 280 ºC for the detector, respectively. Before the analysis, samples of the essential oil were diluted (1% v/v) using n-hexane. A 1:15 split ratio was used for the automatic injection of 1 µL of sample.

### Identification of the oil components

The GC peaks’ mass spectra have been identified by searching libraries (NIST and WILEY). Confirmation of the identification has been achieved by calculating the peaks’ retention indices (RI) in relation to (C6–C22) n-alkanes and comparing the results with published data [22–27]. Peak area percent was used for quantification, and the average of three measurements was presented [28].

### Antioxidant and enzyme inhibitory assays

We examined the antioxidant potential of the essential oils by conducting six distinct spectrophotometric tests. This consisted of the 2,2-azino-bis-3-ethylbenzothiazoline-6-sulphonic acid (ABTS) and 2,2-diphenyl-1-picryl-hydrazyl-hydrate (DPPH) assays, that assess the antioxidants’ aptitude for free radicals’ neutralization. Through the ferric-reducing antioxidant power (FRAP) and cupric-reducing antioxidant capacity (CUPRAC) tests, the reduction activity of the extract was examined. We evaluated the total antioxidant and metal chelating activities using the phosphomolybdenum and ferrozine assays, respectively. All assays, except Metal Chelating Activity (MCA), were standardized with a Trolox standard. MCA was compared in terms of Ethylenediaminetetraacetic acid (EDTA) equivalent per gram of essential oil, as outlined in our earlier publications [29, 30].

### Enzyme inhibition assays

In order to assess the inhibitory effects of the oils under investigation on different enzymes, we employed acetylcholinesterase (AChE), butyrylcholinesterase (BChE), tyrosinase, amylase, and glucosidase. Detailed experimental procedures can be found in our previously published research [29, 31]. In terms of mg of galanthamine equivalents (GALAE) per gram of oil, we measured the inhibition of AChE and BChE, whereas tyrosinase inhibition was expressed as mg of kojic acid equivalents (KAE) per gram of oil. Additionally, inhibition of both α-amylase and α-glucosidase was quantified as millimoles of acarbose equivalents (ACAE) per gram of oil.

### Statistical analysis

The procedures were carried out in triplicate, and differences between the methods were analyzed using ANOVA and Tukey’s test (*p* < 0.05). The statistical analysis was carried out using Graph Pad Prism (version 9.2).

## Results and discussion

### Essential oil yield and composition

**Fig. 1:**
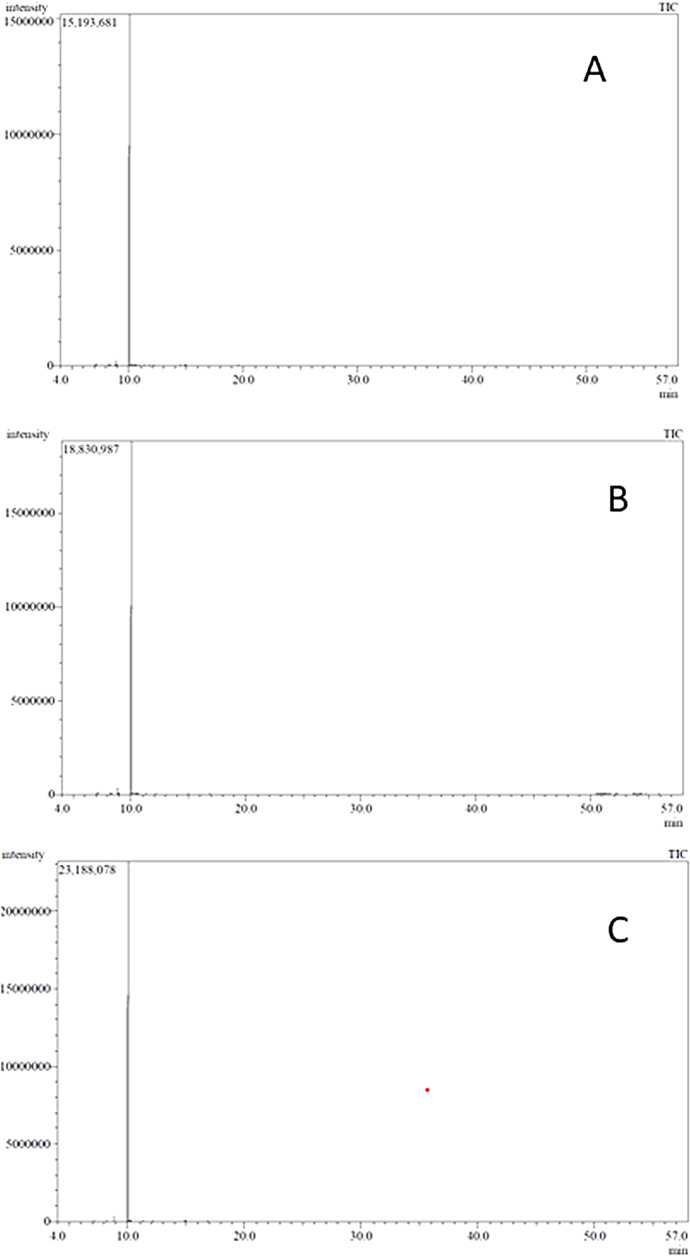
GC-MS chromatograms of (**A**) HD; (**B**) SD; (**C**)MAHD

*C. aurantium* peels essential oils were isolated using three methods; steam distillation (SD), hydrodistillation (HD), and microwave-assisted hydro-distillation (MAHD), the chemical constituents of the oils were analyzed using GC-MS. The GC-MS chromatograms of the essential oil isolated using the three extraction procedures are represented in Fig. 1. This entailed in the identification of a total of six different compounds, which are listed in Table 1. All essential oils obtained by the three extraction techniques were composed mainly of monoterpenoidal compounds. The major constituent obtained by all three methods applied was limonene (98.86% by SD, 98.68% by HD, and 99.23% by MAHD). In our study, some differences were observed in the essential oil composition obtained by the three different extraction techniques. HD has yielded the greatest number of compounds in which two oxygenated monoterpenes namely, linalool and *α*-Terpineol acetate have been obtained with a percent composition of 0.12% and 0.1%, respectively. MAHD has yielded only one oxygenated monoterpene (linalool) with a percent composition of 0.06% from the isolated oil. The SD method showed the absence of oxygenated compounds and resulted in the isolation of only monoterpene hydrocarbons (*α*-pinene, sabinene, and *β*-myrcene). Both *α*-pinene and *β*-myrcene were obtained in all essential oil samples extracted by the three methods, while sabinene was absent in the oil obtained by MAHD as displayed in Table 1.

**Table 1:** Chemical composition of the essential oils using GC/MS analysis

No.	Rt* (min)	Compound^a^ name	Composition %^b^			RI^c^_exp_	RI _rep_	Molecular formula	Identification
			C. aurantium SD	C. aurantium HD	C. aurantium MAHD				
1	7.083	*α*-Pinene	0.27	0.25	0.17	929	932	C_10_H_16_	MS, RI
2	8.259	Sabinene	0.04	0.09	-	968	969	C_10_H_16_	MS, RI
3	8.815	*β*-Myrcene	0.8	0.67	0.54	987	987	C_10_H_16_	MS, RI
4	10.083	Limonene	**98.86**	**98.68**	**99.23**	1029	1029	C_10_H_16_	MS, RI
5	12.155	Linalool	-	0.12	0.06	1096	1095	C_10_H_18_O	MS, RI
6	19.54	*α*-Terpineol acetate	-	0.1	-	1346	1346	C_10_H_20_O_2_	MS, RI
Monoterpene hydrocarbons			99.66	99.69	99.98				
Oxygenated compounds			-	0.22	0.06				
Total identified compounds			99.66	99.91	100				

^a^ Compounds are scheduled according to their elution

^b^ Average of three analyses MS, identification based on mass spectral data

^c^RI, Kovats retention, exp (experimental), rep (reported)

*Rt: Retention time

(-) Unidentified in the samples, (Steam Distillation), HD (Hydrodistillation), MAHD (Microwave-Assisted Hydrodistillation)

The isolation of *C. aurantium* peel EO using the HD method previously reported the identification of twenty chemical compounds [20]. In our current investigation, by employing the same technique only six compounds were identified from the *C. aurantium* peel EO. Our analysis revealed limonene as the major constituent, comprising 98.68% of the isolated EO, which is in line with the previous findings, though the previous study reported a slightly lower abundance of limonene (94.67%). We attribute this variation to geographical differences in the origin of the fruit samples.

EOs isolated from various parts of *C. aurantium* using our study’s isolation methods have consistently shown the presence of limonene, though not as a major constituent. For instance, when isolated from the leaves using the HD method, limonene comprised only 0.71% of the total components [32]. While, major components identified in the leaves’ EO obtained by the HD method were linalool, linalyl acetate, and α-terpineol constituting greater than 73% of the total compounds [33]. Limonene was also detected, comprising 11.67% of the total EO composition from the buds of *C. aurantium* using the SD method, compared to 98.86% obtained in our study from peel EO using the same technique. These findings suggest that EOs isolated from different plant parts using the same technique will lead to different chemical compositions. This is clear also regarding linalool, when extracted using the HD technique in our study, appeared in minor amounts (0.12%), contrasting with the higher composition percentage of 30.62% reported from the *C. aurantium* leaves using the same technique [32].

On the other hand, differences in extraction methods result in variations in the chemical profiles of the EOs. For example, sabinene and *α*-pinene were identified previously from leaves EOs of *C. aurantium* by SD method only and were absent in oils obtained by reflux extraction and ultrasound-assisted extraction technique [33].

From the above, it is concluded that, in our study, although differences in the extraction methods of *C. aurantium* peel oils resulted in differences in the composition of the EOs, limonene remained the major component in all samples. Comparing our findings with the previous reports showed the differences in the essential oils’ composition between different organs of *C. aurantium* even when using the same isolation methods.

### Antioxidant activity

Oxidative stress refers to the disparity between the defensive capabilities of the antioxidant system and the generation of reactive oxygen species. Plant antioxidants are commonly acknowledged as nutraceuticals, serving to eliminate free radicals and functioning as antioxidants [34, 35]. Compounds with reductive properties have traditionally been employed in the food industry to protect products from oxidation [36]. Nonetheless, the current study’s focus lies on their capacity to counteract oxidative stress.

Table 2 represents a comprehensive overview of antioxidant activities associated with the essential oil extracted through three distinct methods namely; HD, SD, and MAHD. The antioxidant activities were measured using various assays including; DPPH, ABTS, CUPRAC, FRAP, PBD, and MCA. When the antioxidant activities were compared across the methods, it was found that the essential oil extracted through the SD method displayed higher antioxidant activity in the DPPH (4.74 mg TE/g) and PBD (49.45 mmol TE/g) assays compared to the HD and MAHD methods. On the other hand, the essential oil extracted through the HD extraction method exhibited higher radical scavenging activity in the ABTS assay (4.09 mg EDTAE/g) and MCA (16.20 mg EDTAE/g). In addition, in DPPH assay, HD (3.44 mg TE/g) and MAHD (2.51 mg TE/g) showed similar ability (*p* > 0.05).

**Table 2:** Antioxidant activities of the three extracted *C. aurantium* essential oils

Methods	DPPH (mg TE/g)	ABTS (mg TE/g)	CUPRAC (mg TE/g)	FRAP (mg TE/g)	PBD (mmol TE/g)	MCA (mg EDTAE/g)
HD	3.44 ± 0.28^b^	4.09 ± 0.83^a^	57.50 ± 2.01^b^	53.92 ± 0.79^b^	42.30 ± 0.03^c^	16.20 ± 0.29^a^
SD	4.74 ± 0.20^a^	1.56 ± 0.13^b^	69.30 ± 1.13^a^	55.84 ± 2.42^ab^	49.45 ± 0.34^a^	15.86 ± 1.98^a^
MV	2.51 ± 0.68^b^	1.24 ± 0.18^b^	70.75 ± 1.02^a^	59.92 ± 1.72^a^	47.89 ± 0.03^b^	12.89 ± 1.59^b^

*Values are reported as mean ± SD of three parallel experiments. DPPH: 2,2-diphenyl-1-picrylhydrazyl; ABTS: 2,2’-azino-bis(3-ethylbenzothiazoline-6-sulfonic acid); CUPRAC: Cupric reducing antioxidant capacity; FRAP: Ferric reducing antioxidant power; PBD: Phosphomolybdenum; MCA: Metal chelating activity. TE: Trolox equivalent; EDTAE: EDTA equivalent. Different letters indicate significant differences in the methods (*p* < 0.05)

This may be attributed to the linalool and *α*-Terpineol contents in this extraction method [37, 38]. However, the MAHD method, while exhibiting the lowest antioxidant activity in most assays, still showed notable values in the CUPRAC (70.75 mg TE/g) and FRAP (59.92 mg TE/g) assays. The results suggest that the choice of extraction method of essential oil significantly influences the antioxidant activity of the volatile oil, with each method yielding distinctive outcomes across various assays. This provides valuable insights for future research, emphasizing the importance of considering these findings when choosing an appropriate extraction method based on the desired antioxidant potential for specific applications. All the tested EOs demonstrated antioxidant activity with some variation. Numerous studies exploring the chemical constituents and bioactivity of different *Citrus* oils have consistently reported robust radical scavenging activity [39, 40]. In the current study, the essential oils (EOs) are primarily comprised of monoterpene hydrocarbons, notably limonene, and the antioxidant properties of limonene have been previously documented [41–43]. Investigations into the chemical composition of essential oils extracted from *C. aurantium* align with our findings, suggesting that the primary components of the oil of the peel are monoterpene hydrocarbons such as limonene [20, 44]. A study conducted by Hsouna et al., reported that *C. aurantium* L. flower extract displayed superior DPPH scavenging activity, with an IC_50_ of 1.85 µg/ml [45]. The EO of *C. aurantium* L. exhibited IC_50_ values of 1040 µg/ml, 1580 µg/ml, and 140 µg/ml in the DPPH assay, reducing antioxidant power, and H_2_O_2_ scavenging respectively [46].

### Enzyme inhibitory activities

In this current investigation, we investigated the therapeutic activity of EOs derived from *C. aurantium* species in Alzheimer’s disease (AD) management, melanogenesis, and diabetes. This evaluation was conducted through the analysis of acetylcholinesterase (AChE) and butyrylcholinesterase (BChE) inhibition, as well as tyrosinase and α-glucosidase inhibition assays. AChE and BChE are enzymes linked to AD development [47], tyrosinase is implicated in pigmentation processes [48], and α-glucosidase is associated with diabetes [49]. Previous studies have demonstrated that bioactive compounds found in plants play a significant role in inhibiting these aforementioned enzymes [50, 51].

Table 3 provides a detailed overview of enzyme inhibition activities of EOs extracted through HD, SD, and MAHD. The enzyme inhibition activities are measured against key enzymes, namely acetylcholinesterase (AChE), butyrylcholinesterase (BChE), tyrosinase, and α-glucosidase.

**Table 3:** Enzyme inhibitory activities of the three extracted *C. aurantium* essential oils

Methods	AChE (mg GALAE/g)	BChE (mg GALAE/g)	Tyrosinase (mg KAE/g)	α-Glucosidase (mmol ACAE/g)
HD	2.47 ± 0.04^a^	2.92 ± 0.12^b^	41.28 ± 3.59^b^	2.62 ± 0.08^a^
SD	2.09 ± 0.01^a^	4.12 ± 0.47^a^	36.76 ± 2.63^b^	2.66 ± 0.06^a^
MV	2.43 ± 0.11^a^	4.21 ± 0.42^a^	83.39 ± 1.03^a^	2.57 ± 0.05^a^

*Values are reported as mean ± SD of three parallel experiments. AChE: Acetylcholinesterase; BChE: Butrylcholinesterase; GALAE: Galanthamine equivalent; KAE: Kojic acid equivalent; ACAE: Acarbose equivalent. Different letters indicate significant differences in the methods (*p* < 0.05)

When comparing enzyme inhibition activities across the extraction methods, it was observed that the essential oils extracted through the HD method exhibited notably higher AChE inhibition at 2.47 mg GALAE/g, closely followed by MAHD measuring 2.43 mg GALAE/g. Moreover, MAHD demonstrated superior BChE inhibition activity, measuring 4.21 mg GALAE/g, while SD essential oils exhibited BChE inhibition at 4.12 mg GALAE/g. Additionally, EO extracted through MAHD displayed the highest anti-tyrosinase activity, recording 83.39 mg KAE/g. In comparison, HD yielded moderate tyrosinase inhibition at 41.28 mg KAE/g, whereas MAHD showed the lowest inhibition at 36.76 mg KAE/g. Furthermore, the essential oils extracted through the SD method exhibited 2.66 mmol ACAE/g, comparable α-Glucosidase inhibition to essential oils extracted through HD, which measured 2.62 mmol ACAE/g. These results highlight the potential of *Citrus* EOsand their components as promising candidates with anti-cholinesterase, anti-melanogenesis, and anti-diabetic activities. They are well-suited for applications in pharmaceuticals targeting neurodegenerative disorders, skin hyperpigmentation, and diabetes.

Previous research showed the enzyme-inhibitory properties of diverse *Citrus* species [52–56]. For instance, *C*. *aurantifolia* and *C*. *aurantium* EOs exhibited AChE inhibition, with respective IC_50_ values of 139.3 and 147.5 µg/mL. While a consistent but comparatively lower bioactivity was noted against BChE, with IC_50_ values ranging from 235.5 to 266.6 µg/mL for both species, respectively [52]. Various monoterpenoids identified in *Citrus* EOs have been investigated for their potential to inhibit cholinesterase [53]. Furthermore, a study involving thirteen different *Citrus* EOs and their volatile flavor constituents revealed significant tyrosinase inhibitory activity [54]. Essential oils from various *Citrus* species were reported to reduce the expression levels of Tyrosinase, suggesting their efficient inhibition of melanogenesis [55]. Moreover, the EO extracted from the peel of *C*. *aurantium* was found to inhibit α-glucosidase activity [56].

## Conclusion

The volatile components from the peel of *C. aurantium* were extracted using three distinct methods: HD, SD, and MAHD. These extracts were subsequently analyzed using GC/MS. Across all three methods, limonene was identified as the major compound, constituting 98.86% in SD, 98.68% in HD, and 99.23% in MAHD extracts. The chemical composition of the oils was varied, attributable to the extraction techniques employed. The antioxidant effects of the essential oils were tested by six different assays; DPPH, ABTS, CUPRAC, FRAP, PBD, and MCA. Results showed that the essential oil extracted using the SD method demonstrated superior antioxidant activity in the DPPH and PBD assays. while the HD extraction method showed higher radical scavenging activity in the ABTS assay and MCA. Comparing the enzyme inhibition activities among different extraction methods for essential oils, essential oils extracted through the HD method showed higher AChE inhibition. While MAHD-extracted oil demonstrated superior BChE and tyrosinase inhibition activities. Additionally, SD essential oils exhibited comparable α-Glucosidase inhibition to HD. Overall, these findings suggest the potential of Citrus EOs and their components for antioxidant, anti-cholinesterase, anti-melanogenesis, and anti-diabetic activities. And not only the plant organ that can affect the biological activity but also the extraction method.

## Data Availability

Data are available upon request from Omayma Eldahshan; oeldahshan@pharma.asu.edu.eg.

## Abbreviations

ABTS: 2,2-azino-bis-3-ethylbenzothiazoline-6-sulphonic acid
ACAE: Acarbose equivalents
AChE: Acetylcholinesterase
AD: Alzheimer’s disease
BChE: Butyrylcholinesterase
CUPRAC: Cupric-Reducing Antioxidant Capacity
DPPH: 2,2-diphenyl-1-picryl-hydrazyl-hydrate
EDTA: Ethylenediaminetetraacetic acid
EO: Essential oils
FRAP: Ferric-Reducing Antioxidant Power
HD: Hydrodistillation
KAE: Kojic acid equivalents
MAHD: Microwave-Assisted Hydrodistillation
SD: Steam distillation
